# Analytic Hierarchy Process (AHP)-Based Aggregation Mechanism for Resilience Measurement: NATO Aggregated Resilience Decision Support Model

**DOI:** 10.3390/e22091037

**Published:** 2020-09-16

**Authors:** Jan Hodicky, Gökhan Özkan, Hilmi Özdemir, Petr Stodola, Jan Drozd, Wayne Buck

**Affiliations:** 1 NATO Headquarters Supreme Allied Commander Transformation, Norfolk, VA 23511, USA; jan.hodicky@act.nato.int (J.H.); wayne.buck@act.nato.int (W.B.); 2 STM Savunma Teknolojileri Mühendislik ve Ticaret A.S., Ankara 06530, Turkey; gokhanozkan@stm.com.tr (G.Ö.); mozdemir@stm.com.tr (H.Ö.); 3 Department of Intelligence Support, University of Defence, 66210 Brno, Czech Republic; 4 Department of Tactics, University of Defence, 66210 Brno, Czech Republic; jan.drozd@unob.cz

**Keywords:** Analytic Hierarchy Process (AHP), NATO resilience, resilience aggregation, resilience measurement, system dynamics

## Abstract

Resilience is a complex system that represents dynamic behaviours through its complicated structure with various nodes, interrelations, and information flows. Like other international organizations NATO has also been dealing with the measurement of this complex phenomenon in order to have a comprehensive understanding of the civil environment and its impact on military operations. With this ultimate purpose, NATO had developed and executed a prototype model with the system dynamics modelling and simulation paradigm. NATO has created an aggregated resilience model as an upgrade of the prototype one, as discussed within this study. The structure of the model, aggregation mechanism and shock parametrization methodologies used in the development of the model comprise the scope of this study. Analytic Hierarchy Process (AHP), which is a multi-criteria decision-making technique is the methodology that is used for the development of the aggregation mechanism. The main idea of selecting the AHP methodology is its power and usefulness in mitigating bias in the decision-making process, its capability to increase the number of what-if scenarios to be created, and its contribution to the quality of causal explanations with the granularity it provides. The parametrized strategic shock input page, AHP-based weighted resilience and risk parameters input pages, one more country insertion to the model, and the decision support system page enhance the capacity of the prototype model. As part of the model, the decision support system page stands out as the strategic level cockpit where the colour codes give a clear idea at first about the overall situational picture and country-wise resilience and risk status. At the validation workshop, users not only validated the model but also discussed further development opportunities, such as adding more strategic shocks into the model and introduction of new parameters that will be determined by a big data analysis on relevant open source databases. The developed model has the potential to inspire high-level decision-makers dealing with resilience management in other international organizations, such as the United Nations.

## 1. Introduction

National and international policymakers have recently been focusing on the resilience concept as one of the most critical sustainability measures for their organisations [1]. As an international military organization, NATO is not that different than these other organisations. In this regard, NATO has developed a prototype resilience model that can provide foresight regarding the NATO resilience changes over time [2]. With the help of the system dynamics modelling and simulation paradigm, the prototype model is capable of evaluating the resilience capacity of a country and dynamically assessing its resilience-based risk. The outcomes of the model comply with NATO’s strategic resilience concept [2]. The strategic resilience concept is an adaptive process in which resilience performance is measured by absorbing strategic shocks with minimal impact while maintaining essential functions at an acceptable level, then recovering functionality at a reasonable time and a reasonable cost. Therefore, a well-integrated system specifically focuses on managing the local consequences of an attack and isolating the event from the overall system function, thus making the adversary’s value proposition unfavourable. Subsequently, the system adapts and increases its capacity to withstand future shocks with reduced vulnerability exposure to the adversary and increased speed of system recovery [3].

Hodicky et al. [2] write about growth potential, such as adding more countries to the model via an appropriate aggregation mechanism and bringing parametrization of strategic shocks (i.e., adding user-specified shock parameters). Accordingly, NATO has developed an aggregated resilience model as an upgrade of the extant prototype by activating the mentioned growth potential. The main objective of the aggregated model employing system dynamics paradigm is replicating key factors and parameters of resilience for a comprehensive and seamless resilience assessment for NATO strategic level decision-makers. The structure of the aggregated resilience model, aggregation mechanism, and shock parametrization methodologies used in the development of the model comprise the scope of this study.

The aggregation mechanism, parametrized strategic shock input structure, and decision support output structure are the prominent features differentiating the aggregated model from the former prototype version. This is all about assigning criticality (importance), magnitude, and effect weights to resilience parameters, countries, and strategic shocks that already exist in the previous version. Because there are relative differences among resilience related variables and parameters in real life, the use of a systematic weight allocation process that can approximate the outcomes of the model to the real-life behaviours was inevitable. While the exact measurement of resilience is hardly possible because of its conceptual complexity, weight assignment to resilience parameters can be one of the initial steps to confirm assumptions [4]. Resilience is a complex system that represents dynamic behaviours through its complicated structure with various nodes, interrelations, and information flows [5]. Such behaviours can be exemplified as the dynamic ones stemming from current and future battlefield environments [6] that are characterized by non-state diplomatic activities, information, and military and economic actions that create secondary and tertiary effects. Even though it is sometimes perceived as ordinary, the weighting process is critical because of its power to mitigate prejudice in decision-making [7]. As such, the Analytic Hierarchy Process (AHP) being a multi-criteria decision-making tool was used for aggregation and parametrization purposes. The AHP-based weight allocation process gives opportunities for the users to create more what-if scenarios. Moreover, with the help of this process, users can enhance the granularity of the model and, thus, increase the quality of causal explanations especially needed by high-level decision-makers.

Similarly, various aggregation approaches and methodologies have been widely used in different areas [8], including the resilience domain, to better understand real-life behaviours and to reflect them within the models. In this regard, a comprehensive aggregation process developed to allocate weights to the indicators in a water supply resilience measurement model [9] and a hierarchical aggregation mechanism used to aggregate different levels of visual patterns to deal with biases stemming from variations and to yield an accuracy [10] can be exemplary. The number of holistic resilience measurement applications where different importance weights are assigned to different resilience dimensions has been increasing [11]. AHP, like other aggregation functions, is broadly used in decision-making processes to combine information [12]. The strength of the AHP stems from its capability of making impartial and sound classifications and its flexibility to aggregate a set of parameters by defining the relative importance of these parameters [13]. As such, model developers and researchers have also been using AHP as an aggregation methodology in the area of risk, safety, and resilience assessment. One example is the AHP-based evaluation system developed to assess habitability performance [14]. Other examples are the application of AHP for risk and sustainability assessment in power plant maintenance [15], urban flood management [16,17], rural water supply management [18], and corporate sustainability [19,20]. Besides the extensive usage of AHP, there are also other discussions about the weaknesses of the methodology. One of the limitations of this method is its weakness to capture uncertainty and, therefore, methods, such as Fuzzy AHP, have been developed to capture uncertainty in decision making [21]. Additionally, it is frequently stated in the literature that there are doubts about the objectivity of the evaluation, as the results are largely based on the subjectivity of decision makers [22]. Another disadvantage of AHP is the loss of information encountered when the good and bad scores given by experts to some criteria compensate each other [23]. Moreover, another weakness is that the difficulty of AHP calculations depends on the number of criteria and alternatives [23]. However, AHP was preferred in this study, because it can solve complex decision-making problems by handling qualitative and quantitative variables together and takes into account the priorities of the decision maker. There are other multiple criteria decision making methods, such as ELECTRE, PROMETHEE, and TOPSIS, which can be listed as alternatives to AHP [23]. ELECTRE and PROMETHEE, which are outranking methods, focus on ranking the alternatives by criterion comparison and TOPSIS determines the distance of each alternative from the goals where the AHP method differentiates from these methods by its full aggregation feature [23]. The AHP-based aggregation mechanism used in this study will replace the arbitrary weight allocation approach that NATO has been using for the resilience evaluation. The introduction of this AHP-based aggregation mechanism to NATO’s resilience management domain makes a substantial difference. The application of AHP methodology in NATO’s resilience management function is both a new feature added to NATO’s existing processes and a contribution to NATO related literature. The AHP methodology proved itself to have high reliability in a comprehensive evaluation system development study by weight determination [24].

The integrative structure of the aggregated model, its capacity to increase the number of what-if scenarios by the help of an aggregation mechanism, and its functional display capability provided by the decision support system page create the real difference. Therefore, the aggregated model helps to enhance the risk management and resilience-building capacities of NATO that is continually experimenting and conducting scenario-driven exercises to prepare for operations. As such, the AHP-based aggregated model is one of NATO’s discovery experiments. The discovery experiment is a test to determine the efficacy of something previously untested in a particular manner. The main objective of this discovery experiment is to evaluate whether the total resilience capacity of NATO countries in a scenario-driven operation can be understood by the help of a data aggregation mechanism via a dynamic model. The outcome of the NATO discovery experiment is the prototype of the aggregated resilience data model. The aggregated prototype model meets the requirement of related NATO units who are looking for a tool to examine the resilience concept, evaluate the effects of strategic shocks on overall resilience capacity in more than one nation operational environment, and understand the interferences of resilience main domains in an integrated and comprehensive manner. Besides the model usage itself, decision makers at NATO operation and strategic level may also benchmark and use the aggregation methodology of the study while generally evaluating military complex systems similar to resilience. All in all, the main study motivation was NATO identified gap in the resilience measurement capability. The developed model has the potential to inspire other international organizations, such as the United Nations, which has also been deeply involved in resilience management.

## 2. Materials and Methods

**Figure 1:**
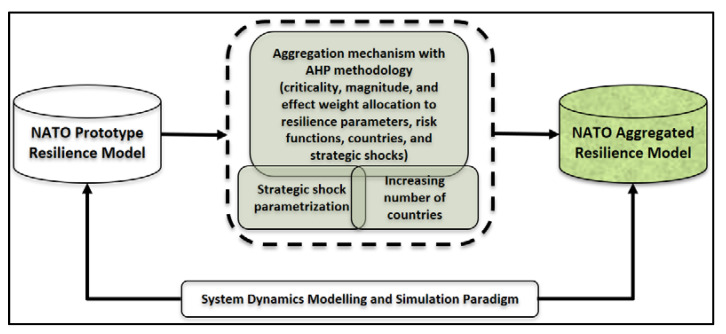
Methodology of the study.

We have used the system dynamics modelling and simulation paradigm to develop both prototype and aggregated resilience models, as depicted in Figure 1. The main rationale behind choosing this paradigm is to quantify NATO’s resilience-related capacities by a structure representing dynamic relations among vague resilience parameters over time in scenario-driven NATO operations and experiments [2]. The system dynamics modelling technique helps us understand and synthesize nonlinear behaviours of complex systems, such as resilience [25,26], by the help of simulation software analysing causal interrelations among the components that make up those systems [2]. In accordance with the system dynamics methodology, we have conducted problem articulation and literature review on concept and measurement techniques of resilience concurrently, identified key resilience variables, made causality and behavioural analysis by building causal loop diagrams, modelled the dynamics of resilience via stock and flow diagrams, and developed user interfaces that consist of input and output dashboards for the prototype and aggregated resilience models. For the further study of methodological steps in a model design, refer to the previously published paper by the authors [2]. We used Stella (Systems Thinking for Education and Research) Architect (version 2) modelling software package during the design and implementation phases of both prototype and aggregated resilience models. The software also has an embedded presentation capacity that we have used during the demonstration of the model at the final workshop with military operational community.

The focal methodology of this study includes overlapping steps, such as developing an aggregation mechanism, parametrizing the strategic shocks, and adding more countries to the prototype model.

### 2.1. Aggregation Mechanism

AHP, which was introduced by Thomas L. Saaty, is the methodology used for the development of the aggregation mechanism. AHP is a multi-criteria decision-making technique using pairwise comparisons made by the judgments of subject matter experts to derive priority scales that measure intangibles in relative terms [27]. The AHP method helps us to transform complex and unstructured problems hierarchically into a series of pairwise comparisons, aims to quantify the priority of the given set, and synthesizes the final judgment to evaluate which alternative has the highest-ranking [13,19]. AHP offers such advantages as the ability to take the preferences of decision-makers into account, the ability to evaluate qualitative and quantitative variables together, and to possess a flexible structure. Additionally, we can check the consistency of the decision maker’s opinions with a specific process within the AHP, thereby reducing bias in decision-making. Pairwise comparison with a hierarchical structure is so powerful for derivation, because only a pair of items is taken and compared on a single property without any concern for other items or properties [28].

**Figure 2:**
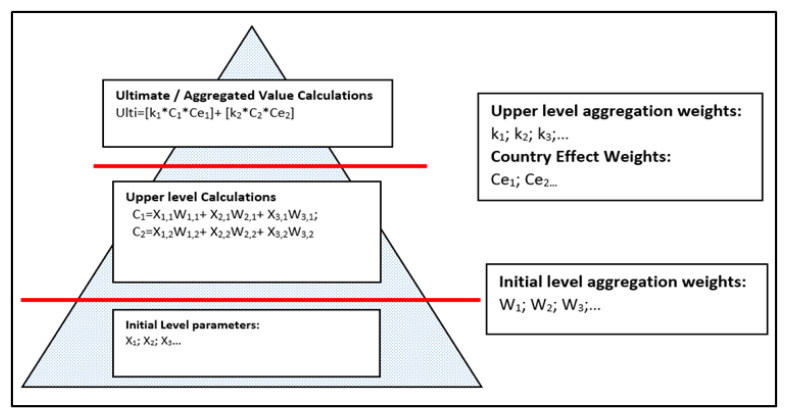
Two-Tier weighting mechanism applied in the description of aggregated resilience and risk in the area of operations.

With AHP, we can execute an organized decision-making process through the following steps: (1) defining the problem (2) structuring the decision hierarchy from top to bottom (goal → objective → criteria → alternative) (3) constructing a set of pairwise comparison matrices and making comparisons by the help of the numbering scale that is provided in Figure 2 (4) obtaining priority weights at each level and calculating final priority value [27].

In Table 1 we have adapted the table from Saaty [27] by only including the values with explicit explanations and excluding intermediate values (2,4,6,8) that have any explanations.

We constructed a two-tier weighting mechanism (Figure 2) through AHP to aggregate values of resilience main domains (civil support to the military, continuity of government, and continuity of essential services) and risk assessment functions (command and control, protection, movement/manoeuvre, and sustain) belonging to different countries.

In accordance with the weighting mechanism, the important weights of each resilience domain and risk function were determined by the subject matter experts with AHP, and then the values of initial level parameters are multiplied by the initial level importance weights, and upper-level values are obtained. The upper-level values are multiplied by upper-level aggregation weights and country effect weights that also have been determined by the experts via AHP. At the end of these calculations, we obtained the ultimate values of the aggregated risk and resilience.

**Figure 3:**
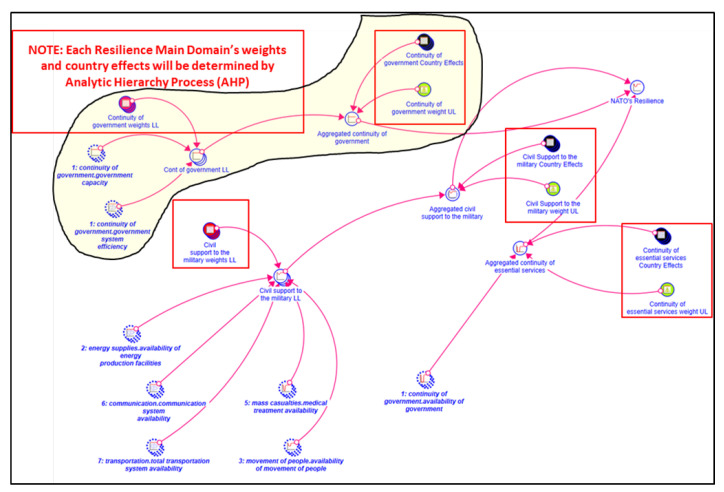
AHP-based aggregation mechanism for resilience main domains.

Figure 3 shows the aggregation mechanism applied for the resilience calculations. As is highlighted in the figure, at the lower level, users have assigned weights for government capacity and government system efficiency parameters to calculate the value of the continuity of government domain. Then at the upper level, users have again determined a weight for the continuity of government, which is one of the resilience main domains to obtain the overall NATO resilience value. In addition to the resilience main domain weights, users have also assigned weights for the countries (i.e., country effects) at the upper level.

Accordingly, we have created new input pages for the resilience main domains and risk assessment functions in the model in order to include importance weights.

**Figure 4:**
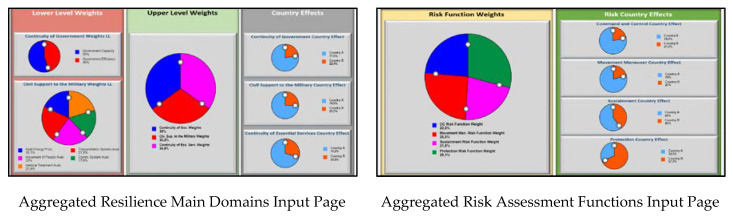
Sample aggregated resilience main domains and risk assessment functions input pages.

Users can insert relevant weights that have been determined through the aforementioned aggregation mechanism into the model by the help of input pages that are depicted in Figure 4. On the left side of the resilience main domains input page, users can define lower level weights for the sub parameters of the resilience main domains where the upper level weights for the resilience main domains can be inserted in the middle of the page and country effects for each resilience main domain are determined on the right side. The aggregated assessment functions input page has two parts where the users can define weights for each risk assessment function and determine country effects for each risk assessment function.

### 2.2. Strategic Shock Parametrization and Adding More Countries to the Model

The strategic shocks (electricity blackout, cyber attack, martial law enforcement, big human movement, state of war, armed conflict) also got their effect weights for each country by following the AHP methodology. We have used these weights in the strategic shock parametrization process.

**Figure 5:**
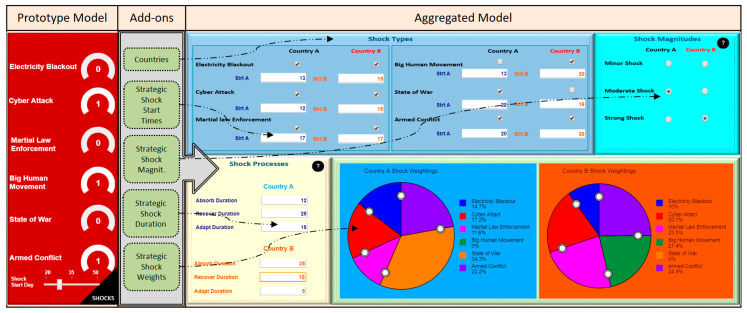
Strategic shock parametrization.

The strategic shocks have been parametrized by adding more features to the strategic shock input page of the model. Figure 5 depicts the before and after snapshots of the shock input pages of the model. The previous version of the shock input page of the prototype model resides on the left side, add-ons applied to the prototype model lays in the middle, and the shock parametrization input page of the aggregated model is shown on the right side. The prototype model column shows the strategic shock types with their status (knob) and a single start day (slider). The add-ons column shows the areas (countries, strategic shock start times, strategic shock magnitudes, strategic shock duration, strategic shock weights), where the changes have been applied to the prototype model. The aggregated model column depicts details of changes that have been implemented on number of countries and strategic shock parameters.

In the prototype model, the users were only able to select relevant shocks (0: no shock; 1: shock) for a country with the same start day for each shock. The extant limited capacity of the prototype model showed us the necessity of approximation of the prototype model to the real world. The add-ons applied to the prototype model include one more country addition to the model, separate start time determination capability for each shock and each country, three different shock magnitude (minor, moderate, strong) assignment capability for each country, determination of shock evolution durations (absorb, recover, adapt) for each country, and AHP-based shock effect weight injection capacity. Although the aggregated model runs with two countries at the moment, it has an infinite capacity to deal with more countries.

With the new features of the input pages that include importance and effect weights determined by the AHP, the aggregated model enables users to create more what-if scenarios with more details and real-world attributes. Additionally, this provides decision-makers more realistic situational awareness.

### 2.3. User Interface

**Figure 6:**
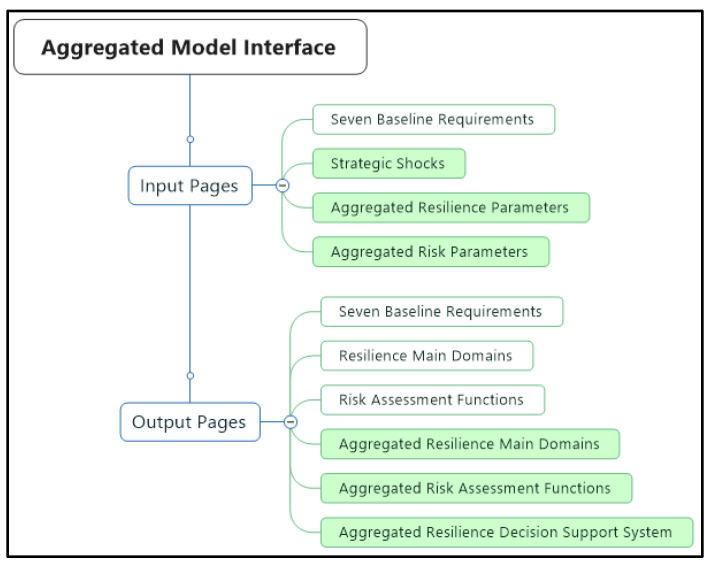
Aggregated model user interface pages.

The aggregated resilience model includes both input and output dashboards, as was the case in the prototype model. We have upgraded the dashboards by developing three input and three output new pages (green color-coded in Figure 6).

**Figure 7:**
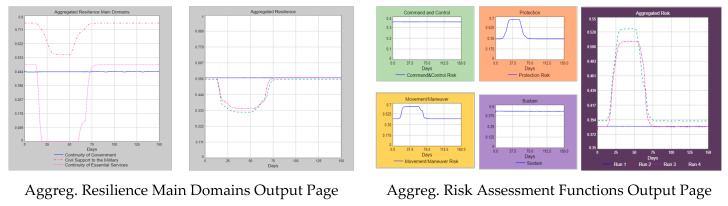
Sample aggregated main domains and risk functions output pages.

The seven baseline requirements input page allows the users to insert values of 15 different parameters for each country. There are various insertion options on the page, such as numeric input, slider, and knob. The newly developed input pages have already been discussed above. Screenshots of the aggregated resilience main domains and aggregated risk assessment functions output pages are provided below (Figure 7). The aggregated resilience main domains output page shows both time-based graphical results of each resilience main domain and NATO’s aggregated resilience upon each simulation run. Alike, the aggregated risk assessment functions output page includes the output values of each risk assessment function and the aggregated risk.

In addition to three separate pages for providing output graphs showing resilience capacities of each baseline requirement and resilience main domain, and level of risk for each risk assessment function [2], the new output pages display the aggregated values of these variables belonging to the countries. Users can see the (time-based) results of each simulation run on the related graphs concurrently. Accordingly, the model provides a benchmarking opportunity for the results that were obtained from different runs.

## 3. Results and Discussion

The user profile of the model consists of military and civilians working at strategic and operational level NATO commands. This profile has been dealing with the resilience phenomenon during the planning and execution of training and exercises and/or real operations. NATO has organized a workshop in order to validate the results of the discovery experiment (AHP-based aggregated model run). The workshop participants have confirmed the applicability of AHP-based aggregation mechanism to deal with the uncertainties and mitigate bias in the decision-making process.

The users employed a fictitious scenario document to set up the model with (artificial) initial values and inject (artificial) shocks into the model scenario during the validation workshop. When the model is run with the baseline scenario (without any shock effect), we get the normal capacity of the baseline requirements, resilience main domains, and the normal risk values. The baseline scenario corresponds to the current situation of Day 0 in the area of operations. When the model is run with shock injections (extraordinary scenario), we see the effects of the shocks on the output values in accordance with the different start times and shock durations.

The most distinctive feature that distinguishes the aggregated model from the prototype one is the aggregated decision support system output page. This page is exclusively designed for the strategic level decision-makers (i.e., FOGOs—Flag and General Officers) to obtain a comprehensive situational awareness about aggregated resilience and risk values of countries by the help of color-coded displays [Red → reduced capacity (0–0.4); Yellow → moderate capacity (0.4–0.7); Green → sufficient capacity (0.7–1.00)].

**Figure 8:**
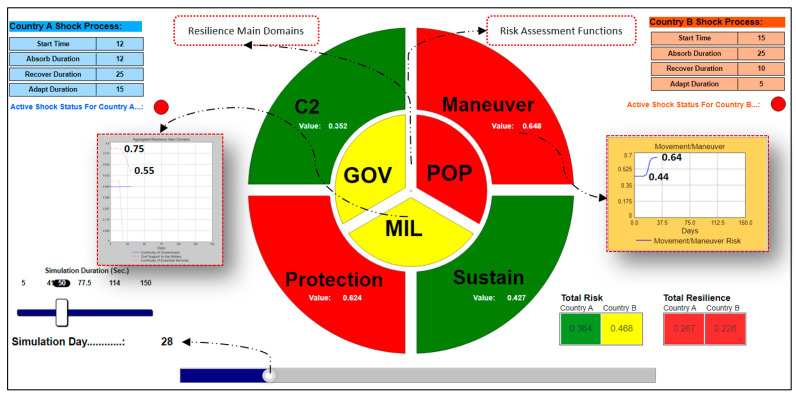
Sample aggregated resilience decision support system page.

Figure 8 depicts a sample decision support system output page. At the top of the page, there are start times and shock process durations of the countries that have been defined by the users in accordance with the scenario document. There are also shock indicators for each country where a yellow small circle turns red upon a shock (i.e., yellow means there is no effective shock within the country; red means that there is an effective shock within the country). There is a slider where users can tune the simulation speed and an indicator that shows the simulation day at the lower-left corner of the page. At the lower right corner, the resilience capacities and the risk levels of the countries are numerically shown with respective colour codes for each country. In the middle of the page, the quartered outer circle represents the aggregated values of each risk assessment function with the corresponding colours. The tripartite inner circle shows the status of each resilience main domain with the aggregated values and corresponding colours. The simulation time slider resides at the very bottom of the page, where users can choose a simulation day and see the aggregated results accordingly.

The above sample decision support system page shows the aggregated resilience and risk situation (operational picture) on the 28th day of the simulation that runs 150 days in total. Strategic shocks started on day 12 in country A and on day 15 in country B, according to this operational picture. Therefore, an extraordinary scenario takes place for both countries. Baseline scenario happens at day 55, the earliest in country B and happens at day 64, the earliest in country A (i.e., all consecutive durations in the shock process are finished in the respective country).

Because the extraordinary scenario is in place for both countries on day 28, both countries have reduced resilience capacities (red color-coded) with the total resilience values of 0.26 and 0.22, respectively. In parallel with the reduced resilience capacities, the risk levels are 0.36 (green color-coded) for country A and 0.46 (yellow color-coded) for country B.

**Figure 9:**
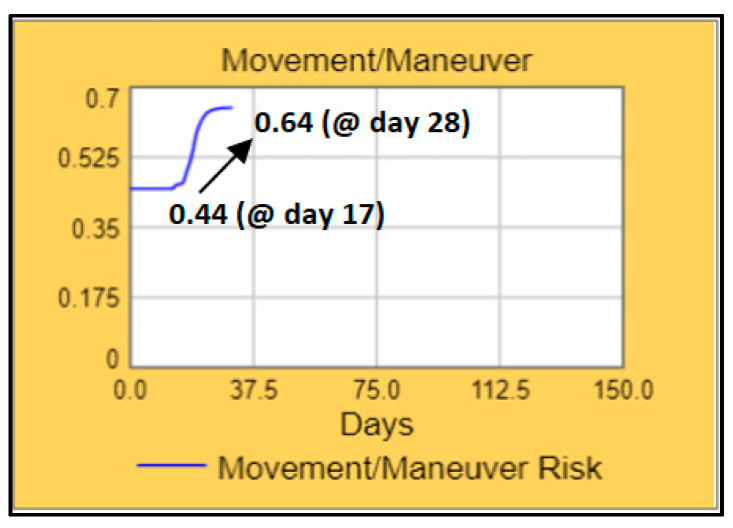
Sample aggregated movement/manoeuvre risk function graph.

When we check the aggregated risk assessment functions that are depicted on the outer circle, C2 (command and control) and sustain risk functions are green color-coded with the values of 0.35 and 0.42 (i.e., the aggregated risk levels in these functions are low and manageable). Comparatively, we see red colour codes for manoeuvre and protection risk functions with the values of 0.64 and 0.62 (i.e., the aggregated risk levels are high and difficult to manage). This decision support output page enables users to see the details of both risk functions and resilience domains by just clicking on the related piece of the circle. The users can see the details of the situation that are producing the colour codes that are seen on the page. If we click on the manoeuvre piece on the outer circle, we see the increase in aggregated risk assessment function (0.44 → 0.64) on the graph covering the duration of Day 0–Day 28, as shown in Figure 9.

**Figure 10:**
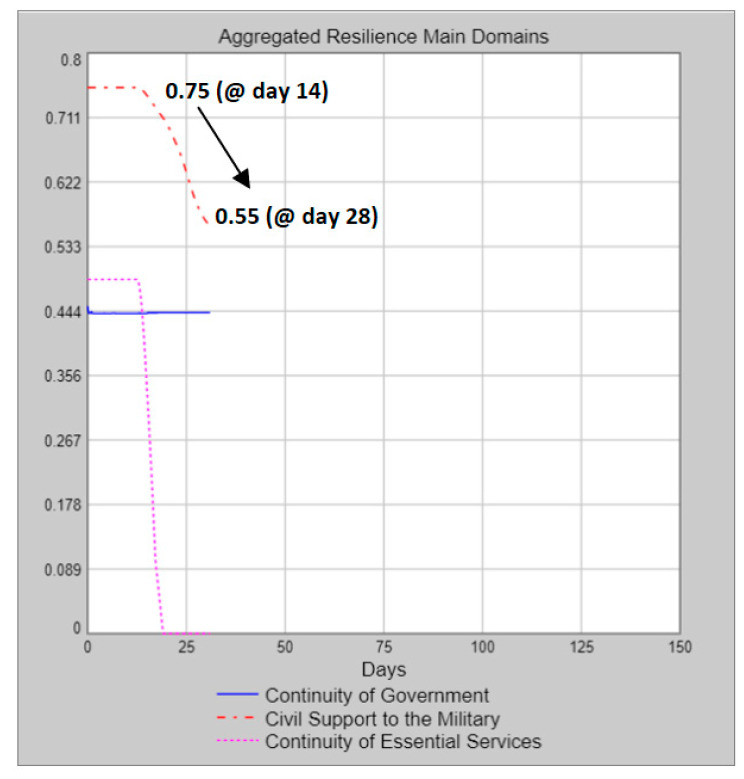
Sample aggregated resilience main domains graph.

Concerning the aggregated resilience main domains, we see yellow colour codes for two domains, such as continuity of government and civil support to the military (i.e., the aggregated resilience capacities are at moderate levels). For the continuity of essential services domain, the colour code is red, meaning that there is a reduced aggregated resilience capacity for this domain. The details of the resilience situation up to Day 28 can be seen on the graph that is opened by a click on the related piece on the inner circle. For example, the decrease in the aggregated resilience value of the civil support to the military domain (0.75 → 0.55) is depicted on the graph presented in Figure 10.

## 4. Conclusions

This study discusses the aggregated resilience model that has been developed in order to provide a means for a comprehensive and seamless resilience assessment for NATO strategic and operational level decision-makers. The scope of this study incorporates the structure of the aggregated resilience model, aggregation mechanism, and shock parametrization methodologies used in the development of the model. The main structure of the model, which is an upgrade of the prototype model, has been constructed using the system dynamics paradigm. We have used the AHP methodology during the aggregation mechanism development and strategic shocks parametrization processes.

The main idea of using the AHP methodology in this study is its power and usefulness in mitigating bias in the decision-making process, its capability to increase the number of what-if scenarios to be created, and its contribution to the quality of causal explanations with the granularity it provides.

The add-ons, such as the parametrized strategic shock input page, AHP-based weighted resilience, and risk parameters input pages, one more country insertion to the model, and the decision support system page fill the development space discussed by Hodicky et.al. [2]. The user interfaces help decision-makers modify the values of selected parameters of different countries concurrently and see the time-based results both in graphs and color-coded displays. Moreover, the model has the potential to enhance the evaluation and foresight capacities of NATO’s communities of interest in the areas of resilience and civil-military cooperation.

The users’ validation workshop exposed that the aggregation, shock parametrization, and the color-coded situation display (decision support system page) capabilities of the model have great potential to make more dynamic and comprehensive evaluations in the area of resilience when compared to the current evaluation capabilities. Moreover, the project results in the form of aggregated resilience model support decision-making as a computational process that progressively eliminates alternatives, thereby reducing uncertainty [29].

The decision support system page stands out as the strategic level cockpit, where the colour codes give a clear idea at first about the overall situational picture and country-wise resilience and risk status. Because NATO high-level managers are used to similar color-codes, this capacity of the model fosters the NATO decision-making process without making a dramatic change in the routines. Additionally, this helps users (strategic level decision-makers) to adapt quickly to use the aggregated model.

Users have discussed potential development opportunities at the validation workshop. Adding more countries and more strategic shocks into the model and introduction of new resilience parameters that will be determined by a big data analysis on relevant open source databases.

## Figures and Tables

**Table 1:** Analytic Hierarchy Process (AHP) numbering scale [27].

Importance Weight	Importance Definition
1	Equally important
3	Moderately important
5	Strongly important
7	Very strongly important
9	Extremely important
